# Cluster analysis to identify elderly people's profiles: a healthcare strategy based on frailty characteristics

**DOI:** 10.1590/1516-3180.2014.1324622

**Published:** 2014-05-20

**Authors:** André Fattori, Ivan Mazivieiro Oliveira, Rosalia Matera de Angelis Alves, Maria Elena Guariento

**Affiliations:** I MD, PhD. Attending Physician, Department of Internal Medicine, School of Medical Sciences, Universidade Estadual de Campinas (Unicamp), Campinas, São Paulo, Brazil; II MD. Resident Physician, Department of Internal Medicine, School of Medical Sciences, Universidade Estadual de Campinas (Unicamp), Campinas, São Paulo, Brazil; III MD. Attending Physician, Department of Internal Medicine, School of Medical Sciences, Universidade Estadual de Campinas (Unicamp), Campinas, São Paulo, Brazil; IV MD, PhD. Professor, Department of Internal Medicine, School of Medical Sciences, Universidade Estadual de Campinas (Unicamp), Campinas, São Paulo, Brazil

**Keywords:** Cluster analysis, Health of the elderly, Disabled people, Frail elderly, Health services for the aged, Análise por conglomerados, Saúde do idoso, Pessoas com deficiências, Idoso fragilizado, Serviços de saúde para idosos

## Abstract

**CONTEXT AND OBJECTIVES:**

The new social panorama resulting from aging of the Brazilian population is leading to significant transformations within healthcare. Through the cluster analysis strategy, it was sought to describe the specific care demands of the elderly population, using frailty components.

**DESIGN AND SETTING:**

Cross-sectional study based on reviewing medical records, conducted in the geriatric outpatient clinic, Hospital de Clínicas, Universidade Estadual de Campinas (Unicamp).

**METHODS:**

Ninety-eight elderly users of this clinic were evaluated using cluster analysis and instruments for assessing their overall geriatric status and frailty characteristics.

**RESULTS:**

The variables that most strongly influenced the formation of clusters were age, functional capacities, cognitive capacity, presence of comorbidities and number of medications used. Three main groups of elderly people could be identified: one with good cognitive and functional performance but with high prevalence of comorbidities (mean age 77.9 years, cognitive impairment in 28.6% and mean of 7.4 comorbidities); a second with more advanced age, greater cognitive impairment and greater dependence (mean age 88.5 years old, cognitive impairment in 84.6% and mean of 7.1 comorbidities); and a third younger group with poor cognitive performance and greater number of comorbidities but functionally independent (mean age 78.5 years old, cognitive impairment in 89.6% and mean of 7.4 comorbidities).

**CONCLUSION:**

These data characterize the profile of this population and can be used as the basis for developing efficient strategies aimed at diminishing functional dependence, poor self-rated health and impaired quality of life.

## Abstract (pt)

**CONTEXTO E OBJETIVOS:**

O panorama social do envelhecimento da população brasileira promete transformações significativas na atenção à saúde. Por meio da estratégia de análise por conglomerados *(cluster)*, procurou-se descrever demandas de cuidados na população de idosos, utilizando componentes da fragilidade.

**TIPO DE ESTUDO E LOCAL:**

Estudo transversal baseado em revisão de registros médicos, realizado no Ambulatório de Geriatria do Hospital de Clínicas da Universidade Estadual de Campinas (Unicamp).

**MÉTODOS:**

Foram analisados 98 idosos usuários deste ambulatório por meio da análise de conglomerados, utilizando instrumentos para a avaliação do status geriátrico global e das características de fragilidade.

**RESULTADOS:**

As variáveis que mais fortemente influenciaram a formação dos conglomerados foram idade, capacidades funcional e cognitiva, presença de comorbidades e número de medicamentos utilizados, sendo o modelo com quatro agrupamentos estatisticamente mais robusto. Podem-se identificar três grupos principais de idosos, o primeiro de bom desempenho cognitivo e funcional, porém com elevada prevalência de comorbidades (idade média 77.9 anos, 28.6% com déficit cognitivo, média de 7.4 comorbidades); outro caracterizado por idade mais avançada, maior comprometimento cognitivo e maior dependência (idade média 88.5 anos, 84.6% com déficit cognitivo, média de 7.1 comorbidades); e ainda outro de menor idade, pior desempenho cognitivo e maior número de comorbidades, porém funcionalmente independentes (idade média 78.5 anos, 89.6% com déficit cognitivo, média de 4.9 comorbidades).

**CONCLUSÃO:**

Esses dados caracterizam o perfil dessa população e servem como substrato para o desenvolvimento de estratégias cujos objetivos sejam diminuir a dependência funcional, autopercepção ruim de saúde e comprometimento da qualidade de vida.

## INTRODUCTION

According to data published by the United Nations Population Fund, by 2050, 22% of the world population is projected to be 60 years of age or over, and 16% is likely be 65 years of age or over.^1^ An estimate from the Brazilian Institute for Geography and Statistics has shown that the percentage of the Brazilian population aged sixty years or over grew from 9.1% in 1999 to 11.3% in 2009. In 2000, 1.8 million people in Brazil were aged 80 years or over. However, by 2050 this figure may reach 13.7 million.^2^ Currently, the resources available to cater for the demands of this age group are limited, both quantitatively and qualitatively, in terms of facilities, specific programs and appropriate human resources.^1^

As populations age, one of the greatest challenges for healthcare policy is to strike a balance between support for self-care (people looking after themselves), informal support (care from family members and friends) and formal care (health and social services).^3^ ^,^ ^4^

Against this background, non-communicable chronic diseases (NCCD), which increase in incidence and prevalence as a population ages, are an important cause of mortality and morbidity both in developed and in developing countries, thus leading to worse quality of life for elderly people.^3^ ^,^ ^5^ ^,^ ^6^ Furthermore, such diseases are associated with a high financial cost and with functional and cognitive deficiencies.^3^ ^,^ ^7^ ^,^ ^8^ NCCDs currently account for approximately 70% of the disabilities among the elderly in Brazil, where the disability-free life expectancy is 59.8 years, 12 years less than the total life expectancy.^2^ Since functional losses tend to become more accentuated with advancing age, the number of people in older age groups in which there is a greater prevalence of functional decline and a greater demand for care is expected to increase.^7^ ^,^ ^9^ ^-^ ^11^

Based on this information, healthcare for the geriatric population must be planned using data that allow risks, possible actions and estimates of costs to be evaluated concisely and objectively.^8^ Ideally, these instruments need to be both easy to apply and suitable for assessing the vulnerabilities of this population, such as their functional and cognitive disabilities, the prevalence of chronic degenerative diseases and the need for pharmacotherapeutic support.^12^ Hence, public policies for care for the elderly can benefit greatly from using constructs to evaluate clinical, psychological and social characteristics that simultaneously represent the major geriatric syndromes and the more recently studied aspects of senescence and senility.^8^ ^,^ ^12^ ^-^ ^14^

The purpose of this study was to identify groups of elderly people at the geriatric outpatient clinic of Hospital de Clínicas, State University of Campinas (Universidade Estadual de Campinas, Unicamp), using a cluster analysis approach based on components associated with frailty syndrome. Through evaluating this type of profile, it might be possible to draw up strategies of greater efficiency for preventing and treating diseases and intercurrent events affecting these elderly individuals, thereby ensuring that they have greater functional independence and autonomy, and to provide guidance and assistance to families and caregivers about the type of care that should be provided for these patients.^3^ ^,^ ^13^ ^,^ ^15^

## OBJECTIVE

The objective of this study was to delineate profiles of elderly people with NCCDs by using the strategy of cluster analysis, based on cognitive status, depressive symptoms, functional capacity to perform activities of daily living (ADLs) and instrumental activities of daily living (IADLs), and the quantity of comorbidities.

## METHODS

The data came from the medical records of patients registered between August 2007 and January 2008. All patients aged over 65 years who were attended in the geriatric outpatient service were eligible for the study. Out of the initial 198 records selected, 98 cases were included in the study and 91 were excluded based on the following exclusion criteria: death, records not located or records without two or more of the variables used to form clusters.

Information regarding gender, age, education level, recorded comorbidities and use of medication reported at the last consultation were collected with the aid of a standardized questionnaire used in the service for overall geriatric assessment. Data routinely recorded during geriatric assessments were gathered from the records, i.e. data from the ADL scale adapted from Katz et al.,^16^ the IADL scale adapted from Lawton and Brody^17^, the Geriatric Depression Scale (GDS) adapted from Yesavage et al.^18^ and the Mini-Mental State Examination (MMSE)^16^ ^-^ ^19^ adapted from Folstein et al.^20^ These scales are used regularly on all the elderly individuals followed up at this service, initially during the first visit and then annually by physicians trained by the team that supervises care provision in the Geriatric Outpatient Unit. Comorbidities and medications used were recorded numerically. Data relating to ADLs and IADLs were assigned scores from 0 to 6 and 0 to 7, respectively; one point was assigned to each activity that an individual was unable to perform independently (the higher the score was, the greater the degree of functional dependence for the parameters evaluated was). Depressive symptoms were evaluated by means of the GDS, such that a cutoff of ≥ 6 was taken to be suggestive of depressive symptoms. For the categorical analysis on MMSE scores, the cutoff was adjusted according to the number of years of formal education, i.e. a minimum expected score of 24 was used for elderly individuals with one or more years of education, and a score of 19 for illiterate individuals. 

To describe the sample profile, tables were drawn up showing frequencies of categorical variables and descriptive statistics for continuous variables. The categorical variables were compared between the groups using the chi-square test or, when necessary, the Fisher exact test (for expected values of less than 5). For the numerical variables, the Mann-Whitney test (two groups) and Kruskal-Wallis test (three or more groups) were used. The relationship between the numerical variables was determined using Spearman's correlation coefficient. A significance level of 5% (P < 0.05) was defined for all the statistical tests.

The following variables were used to study the cluster profiles in the sample: gender, age, number of diseases, number of medications, functional status, cognitive status and depressive symptoms. Partition cluster analysis was then used to establish the number of groups that should be formed. Using the distances between individuals and between groups for each of the above variables, clusters were formed in such a way that the distances between members of the same cluster were as small as possible and the distances between the centers of the clusters as large as possible. Because of the size of the sample, the hierarchical method proved to be unviable. The partition method was therefore chosen, and it was decided a priori to create 2, 3 and 4 clusters.

## RESULTS

Although the differences were not statistically significant, there was a predominance of men among the elderly people aged between 60 to 79 years (58.8% men versus 41.2% women) and a predominance of women among the most elderly (≥ 80 years) (41.2% men versus 57.8% women; P = 0.117) (Table 1).

**Table 1:** Descriptive analysis on the general population and comparative analysis between the two genders of the numerical variables assessed in this sample of elderly people. Outpatient Geriatric Service (n = 98)

Mean ± SD	General	Male	Female	P-value*
Age (years)	79.6 (± 7.35)	77.8 (± 7.01)	80.5 (± 7.41)	0.051
Education level (years)	2.2 (± 2.98)	3.1 (± 3.94)	1.9 (± 2.49)	0.465
Number of comorbidities	6.5 (± 2.26)	6.0 (± 1.93)	6.8 (± 2.40)	0.186
Number of medications	5.7 (± 2.38)	5.4 (± 2.31)	5.9 (± 2.41)	0.257
Geriatric Depression Scale (score)	5.2 (± 3.46)	4.7 (± 2.71)	5.5 (± 3.78)	0.414
Mini-Mental State Examination (score)	20.7 (± 5.18)	22.1 (± 4.32)	20.2 (± 5.45)	0.108
Activities of Daily Living impairments	0.6 (± 1.16)	0.3 (± 1.09)	0.7 (± 1.17)	0.006
Instrumental Activities of Daily Living impairments	2.2 (± 2.60)	1.0 (±2.11)	2.8 (±2.64)	< 0.001

SD = standard deviation

* comparison between genders using the Mann-Whitney test

The majority of the sample (88.3%) consisted of individuals who had had up to four years of formal education or were illiterate. There was no statistically significant difference between the genders. Only seven individuals had had five or more years of formal education.

In the total population, the mean number of diseases and mean number of medications used were 6.5 ± 2.26 and 5.7 ± 2.38 per person, respectively; no statistically significant differences in these figures were observed when the population was stratified according to age group or gender. Spearman's correlation coefficient revealed a positive correlation between the number of diseases and the number of medications prescribed (P = 0.0013) (Tables 1 and 2). When the diseases were analyzed in terms of their classification in the International Classification of Diseases, 10^th^ edition (ICD-10), to identify the frequency with which organic systems were affected individually, cardiovascular diseases had the highest prevalence (29.69%), followed by endocrine and metabolic disorders (of which diabetes mellitus accounted for 17.19%), diseases of the osteoarticular system (of which osteoarthritis accounted for 12.97%) and impaired sight and hearing (8.44%). The most frequently used medications were antihypertensive drugs (combinations of beta blockers, calcium channel blockers, angiotensin-converting enzyme inhibitors and angiotensin receptor blockers; 19.51%), followed by diuretics (12.16%), antiplatelet drugs (7.88%), drugs for osteoporosis treatment (7.88%) and agents for cholesterol control (5.19%) (data not shown in the tables).

**Table 2:** Analysis of the correlation between age and the characteristics assessed in the population sample. Outpatient Geriatric Service (n = 98) (numerical variables, Spearman's correlation coefficient)

Age – Spearman’s correlation coefficient			
	r	P-value	n
Weight	-0.35660	0.0004	96
Height	-0.24288	0.0197	92
Comorbidities	0.02703	0.7916	98
Medications	-0.17547	0.0840	98
GDS	-0.12477	0.264	82
MMSE	-0.24238	0.0199	92
ADL	0.08571	0.4014	98
IADL	0.25744	0.0105	98

r = Spearman's correlation coefficient

**significance level:** < 0.05

n = number of subjects

GDS = Geriatric Depression Scale

MMSE = Mini-Mental State Examination

ADL = Activities of Daily Living

IADL = Instrumental Activities of Daily Living

Cognitive deficit, functional capacity and the presence of depressive symptoms were evaluated using the MMSE, ADL/IADL and GDS, respectively. Although women had a greater number of depressive symptoms (≥ 6 items on the scale), and cognitive deficit adjusted for education was more common in this group, this difference was not statistically significant: 46.30% of the women presented depressive symptoms, versus 39.29% of the men (P = 0.544); and 62.90% of the women presented cognitive impairment, versus 50% of the men (P = 0.239). The recorded prevalence of cognitive deficit was high (in 58.7% of the population sample), and although there was neither a numerical nor a categorical association between the data for cognition and for age, Spearman's index revealed a negative correlation between MMSE and age, i.e. MMSE scores decreased as age increased (r = -0.242 and P = 0.0199) (Table 2).

The data for functional assessment, which were estimated using the ADL and IADL scales and divided into categories, revealed greater functional impairment among women, such that 5.88% of the women and 12.50% of the men presented two or more ADL impairments (P ADL = 0.014) and 8.82% of the women and 12.50% of the men presented four or more IADL impairments (P IADL < 0.001). Even when the data were evaluated as numerical variables, ADL and IADL deficits were observed among the elderly women, such that there were 0.73 and 0.32 impaired ADLs among women and men, respectively (P ADL = 0.006) and 2.84 and 1.09 impaired IADLs among women and men, respectively (P IADL < 0.001). Another component that affected functional status was age, since there were more elderly individuals with four or more impaired IADLs among the patients aged 80 years or older (43.14%) than among the patients in the 60 to 79-year age range (14.89%) (P = 0.009).

Cluster analysis showed that the model with four groups yielded the smallest distance between individuals with similar characteristics and the greatest inter-group distance (R^2^ = 0.318). The variables that contributed the most to the formation of clusters were the numbers of impaired functions determined on the ADL and IADL scales, cognitive impairments and the number of medications used.

Table 3 shows the details of how the groups were formed. Cluster 1 consisted of a few less elderly individuals in whom cognitive deficits were highly prevalent, who had a high degree of functional loss on the ADL and IADL scales, had an intermediate number of diseases and used an intermediate number of medications. Cluster 2 was made up of less elderly individuals in a younger age group who presented better results from cognitive assessment, with preserved ADLs, but had a large number of comorbidities and used a large number of medications (mean age 77.9 years, cognitive impairment in 28.6% and mean comorbidity score of 7.4). Cluster 3 consisted mainly of older individuals using multiple medications who had a higher frequency of cognitive deficits, little functional loss on the ADL scale, significant functional loss on the IADL scale and an intermediate number of diseases (mean age 88.5 years, cognitive impairment in 84.6% and mean comorbidity score of 7.1). Cluster 4 was composed of fewer elderly individuals in whom cognitive deficits were very common, who presented little functional loss on the ADL and IADL scales and who neither had many comorbidities nor used many medications regularly (mean age 78.5 years, cognitive impairment in 89.6% and mean comorbidity score of 7.4). 

**Table 3:** Groups of elderly people categorized by means of the cluster analysis, and the results from continuous variables characterized in the model. Outpatient Geriatric Service (n = 98)

		Cluster 1 (CL1)		Cluster 2 (CL2)		Cluster 3 (CL3)		Cluster 4 (CL4)	P-value*
	n	Mean (SD)	n	Mean (SD)	n	Mean (SD)	n	Mean (SD)	
Age (years)	4	79.2 (± 8.06)	49	77.9 (± 6.09)	13	88.5 (± 4.58)	32	78.5 (± 7.64)	< 0.001
ADL (no.)	4	5.0 (± 0.82)	49	0.3 (± 0.52)	13	1.3 (± 1.12)	32	0.1 (± 0.34)	< 0.001
IADL (no.)	4	6.7 (± 0.50)	49	1.6 (± 1.95)	13	6.0 (± 1.58)	32	1.0 (± 1.90)	< 0.001
Comorbidities (no.)	4	6.5 (± 0.58)	49	7.4 (± 1.96)	13	7.1 (± 2.82)	32	4.9 (± 1.65)	< 0.001
Medications (no.)	4	5.75 (± 3.40)	49	6.73 (± 2.07)	13	6.3 (± 1.50)	32	3.84 (± 1.90)	< 0.001

* comparison of the variables between the four clusters using the Kruskal-Wallis test

**Significant differences:** (Dunn's post-hoc test; P < 0.05): Age (CL1?CL3; CL2?CL3; CL?CL4)

ADLs (CL1?CL2; CL1?CL3; CL1?CL4; CL2?CL3; CL3?CL4)

**IADLs:** (CL1?CL2; CL1?CL4; CL2?CL3; CL3?CL4)

**Comorbidities:** (CL2?CL4)

**Medication:** (CL2?CL4; CL3?CL4)

ADLs = Activities of Daily Living

**IADLs:** = Instrumental Activities of Daily Living

## DISCUSSION

Recognition of the fact that a population is aging results in the need for practices associated with "healthy aging" and allows public health authorities to analyze possible policies for intervention, disease prevention and maintenance of wellbeing. The scope of such practices and policies is not limited exclusively to the biological aspect of care but prioritizes multidisciplinary attention to quality of life, mental health, social support networks and the process of adapting to the circumstances associated with the transition to old age.^3^ ^,^ ^4^ ^,^ ^15^ ^,^ ^21^ ^,^ ^22^

The development of disabilities at the end of life has been well characterized, and recognition of functional losses as an unfavorable outcome to be prevented shifts the focus from disease-centered care, which is usually provided in the form of specialized medical care, to global care aimed at ensuring maintenance of wellbeing.^3^ ^,^ ^8^ ^,^ ^9^ ^,^ ^11^ ^,^ ^13^ ^,^ ^15^ ^,^ ^21^ Thus, although development of profiles identifying groups that are at most risk of functional impairment may not be of immediate relevance to family members, physicians or caregivers, whose attention is focused on the individual, it allows strategies for care of the elderly to be planned.

More recently, studies have correlated vulnerability and functionality to the concept of frailty among the elderly.^23^ ^-^ ^25^ ^,^ ^47^ From the perspective of geriatrics and gerontology, frailty constitutes an important clinical condition characterized by homeostatic imbalance and loss of physiological reserves, resulting from the action of multiple stressors and predisposing social and genetic factors.^23^ ^,^ ^26^ ^,^ ^28^ There is no definitive consensus about the standard methodology for classifying frailty. Fried at al. proposed a strictly biological model that used the criteria of unintentional weight loss, exhaustion, decreased hand grip strength, low level of physical activity and reduced gait speed.^28^ Another proposal for classifying frailty is based on the frailty index introduced by Rockwood et al.^23^ Additionally, this index consists of a multidimensional model in which the phenotype is considered to be the sum of multiple deficits, including clinical disorders, disabilities, psychological disorders, cognitive disorders, imbalance and sensory abnormalities. This concept was the basis for supporting the objective of the present study.^23^ ^,^ ^24^ ^,^ ^29^ ^,^ ^30^ Independently of the criteria used to classify the phenotype, studies have shown higher rates of mortality, functional loss and adverse clinical outcomes in this group. However, variations in the prevalence of frailty and in the risk factors associated with this condition in different populations have confirmed the importance of analyzing regional particularities that lead to changes that have a clinical impact on the way the aging process takes place.^22^ ^,^ ^31^ ^-^ ^33^

Cluster analysis is a useful tool for identifying profiles associated with multifactorial processes. Although few studies have used this methodology to investigate the clinical profiles of elderly individuals, some groups have validated the frailty phenotypes in the elderly proposed by Fried et al.^27^ ^,^ ^28^ and Rockwood et al.^29^ ^,^ ^30^ using different analytical criteria in longitudinal studies; the parameters used in these studies included depressive symptoms, grip strength, cognition and self-perception of health.^12^ ^,^ ^34^ ^-^ ^36^ The ability to directly modify the criteria used in stratifying the profiles allows the methodological design to be adjusted to suit the focus of the analysis.^35^ ^,^ ^36^ ^,^ ^38^ In the present study, the components proposed for the clusters analyzed used parameters that can be easily applied in a clinical setting, and allow overall geriatric status and the indexes related to frailty in the elderly to be assessed at the same time. This is an approach used to plan prevention or intervention measures aimed at reducing unfavorable clinical outcomes.^12^ ^,^ ^38^ The analysis therefore included variables described in connection with senility and frailty (age, cognitive abilities assessed on the MMSE scale, presence of depressive symptoms measured on the GDS scale, ADL and IADL functional statuses, number of comorbidities and number of drugs used regularly) to define significant profiles.^9^ ^-^ ^11^ ^,^ ^22^ ^,^ ^23^ ^,^ ^39^

The results revealed that there were clusters made up of only four individuals, a number that was too small to allow inferences to be made correctly. Clusters 2, 3 and 4 were considered to present greater significance, since the number of elderly individuals in these clusters was higher. 

Cluster 2 was the largest grouping, and was made up of individuals with better functional and cognitive status but high prevalence of comorbidities. Although the large number of diseases in this group of patients can mean greater expenditure on health support and a need for more frequent clinical assessments, these individuals retained their ADL and IADL functional independence.

Cluster 3 was characterized by older individuals, greater prevalence of females and an intermediate number of comorbidities, but was marked by cognitive and functional impairment. The individuals in this cluster had higher IADL disability scores but relatively well-preserved ADL functioning. This difference in functional capacity measured on the two scales was a specific characteristic of this group. It is not unlikely that the loss of the ability to perform more complex functions (IADLs) independently is related to progression of limitations inherent to old age, while the ability to perform more fundamental tasks, such as those represented by ADLs, was retained. 

Lastly, cluster 4 consisted of elderly individuals who, although exhibiting poor cognitive performance, had smaller numbers of comorbidities and retained their independence in relation to ADLs and IADLs. 

This study identified groups with specific care requirements that would reduce the limitations and loss of quality of life resulting from both disabilities and cognitive deficit.^15^ ^,^ ^40^ ^-^ ^43^ Maintenance of functional status is related to subjective wellbeing, self-efficacy and greater resilience and should take priority over primarily clinical and medical approaches. Studies have shown that functional losses can be reversed or minimized through approaches that modify living habits and training for ADLs and IADLs, with positive consequences for self-perception of health.^14^ ^,^ ^15^ ^,^ ^21^ ^,^ ^40^ ^,^ ^41^ ^,^ ^43^ ^-^ ^45^

It should also be noted that the presence or otherwise of a cognitive deficit should be taken into account when planning care actions, as this is an important parameter in overall geriatric assessment.^41^ ^,^ ^42^ ^,^ ^46^ Recent studies have shown that, regardless of whether cognitive impairment is a cause or an effect, it is associated with frailty among the elderly.^39^ ^,^ ^42^ The large percentage of individuals (59.0%) with an MMSE score of either less than or equal to 19 among illiterate members of the study population or individuals with less than one year of education, or less than or equal to 23 among those with more than one year of education, suggests that cognitive decline may exist among this elderly population and that there is a need for additional neuropsychological assessment. There is a known inverse relationship between age and cognitive function, i.e. the older the age group is, the lower the MMSE score will be. The prevalence of the syndrome of dementia tends to increase with age, ranging from 1% to 5% at 65 years of age and increasing to 20% at 80 years and 45% in individuals over the age of 80. Furthermore, it is important to recognize the initial symptoms of cognitive impairment before starting pharmacological treatment and rehabilitation procedures.^15^ ^,^ ^46^

One possible criticism of the present study is that although a limited number of years of education does not have immediate functional implications, it is a limiting factor in cognitive assessments on these individuals. Irrespective of this, identification of groups with preserved physiological function, but with cognitive deficit, allows these groups to be characterized and the requirements for rehabilitation and maintenance of functional capacity in these groups to be predicted. For clusters 3 and 4, it is important to take into consideration any requirements that elderly individuals with disabilities relating to these deficits may have with regard to support from family members and caregiver networks.

Another weakness of this study was its cross-sectional observational nature and the fact that it was based on an analysis of medical records. The lack of longitudinal follow-up of the population sample meant that the progress of each patient's condition could not be analyzed. A follow-up would have made it possible to identify clinical outcomes within each group and intergroup mobility resulting from increasing patient age and from adaptation and intervention measures. Further studies are required to elucidate these issues.

In this study, we describe cluster analysis as a methodological approach based on characteristics associated with major geriatric syndromes for recognizing at-risk groups in a sample. This methodology is proposed as a tool for health managers to use in planning healthcare interventions. Ideally, further longitudinal studies repeating the strategy described here and using the frailty phenotype defined by Fried et al. should be carried out.^28^

## CONCLUSION

These data characterize the profile of this population and can be used as the basis for developing efficient strategies aimed at diminishing functional dependence, poor self-rated health and impaired quality of life.
